# Prognostic value of light reflex pupillometry in Alzheimer’s disease – a longitudinal cohort study

**DOI:** 10.1186/s13195-025-01794-8

**Published:** 2025-07-11

**Authors:** Mathias Holsey Gramkow, Frederikke Kragh Clemmensen, Ulrich Lindberg, Ian Law, Otto Mølby Henriksen, Gunhild Waldemar, Steen Gregers Hasselbalch, Kristian Steen Frederiksen

**Affiliations:** 1 Danish Dementia Research Centre, Department of Neurology, Copenhagen University Hospital - Rigshospitalet, Inge Lehmanns Vej 8, Copenhagen, DK-2100 Denmark; 2 Functional Imaging Unit, Department of Clinical Physiology and Nuclear Medicine, Copenhagen University Hospital - Rigshospitalet, Copenhagen, Denmark; 3 Department of Clinical Physiology and Nuclear Medicine, Copenhagen University Hospital - Rigshospitalet, Copenhagen, Denmark; 4 Department of Clinical Medicine, Faculty of Health and Medical Sciences, University of Copenhagen, Copenhagen, Denmark

**Keywords:** Prognosis, Quantitative pupillometry, Alzheimer’s disease, Bedside prognostication

## Abstract

**Background:**

The pupillary light reflex (PLR) has been indicated as a biomarker in Alzheimer’s disease and may be suitable for easy prognostication. We sought to evaluate the prognostic potential of quantitative light reflex pupillometry (qLRP) in early AD.

**Methods:**

At baseline, 3-months, 12-months and at 18/24-months follow-up (FU), we carried out qLRP with a hand-held pupillometer (PLR-3000, NeurOptics^®^). We assessed clinically evaluated progression, Mini Mental-State Examination (MMSE), visual progression on [^18^F]FDG-PET and Clinical Dementia Rating Sum-of-Boxes (CDR-SoB) at 1-year FU. Logistic and linear regression models were fitted with baseline qLRP and the short-term dynamic qLRP change from baseline visit to 3 months as predictors with adjustment for age and sex. We evaluated logistic regression models by the cross-validated area under the receiver operating curve (AUC).

**Results:**

A decrease in resting pupillary diameter was associated with a higher risk of clinically evaluated progression (odds ratio 4.3, 95% confidence interval (CI): 1.2–16.9) and predicted this outcome with an associated AUC of 0.65. Less relative pupillary change after light stimulus measured at baseline was associated with cognitive decline on MMSE (β = -5.1, 95% CI: -1.6 – -8.6, *p* = 0.004) and a decrease in this variable from baseline–3 months could predict visual progression of [^18^F]FDG-PET (AUC 0.63). There were no significant associations between qLRP and changes in the CDR-SoB, although estimates were in the same direction.

**Discussion:**

qLRP holds promise as a prognostic, bedside, digital biomarker in Alzheimer’s disease, possibly reflecting changes in the arousal state as a disease progression marker. Our results await confirmation in larger cohorts.

**Supplementary Information:**

The online version contains supplementary material available at 10.1186/s13195-025-01794-8.

## Introduction

There exist no approved prognostic markers for Alzheimer’s disease (AD) and current candidates may be too cumbersome for patients to undergo or are not available outside highly specialized centers [1]. The disease course of AD is highly variable [2] and patients and physicians are thus left without important information at the early stages. This prognostic information could potentially be used for advance care planning, treatment selection and for directing clinical follow-up. As disease-modifying treatments are becoming available as treatment options [3, 4] markers to inform treatment decisions are urgently needed both from a patient and clinician perspective [5]. Digital biomarkers such as light reflex pupillometry offer unobtrusive continuous monitoring of symptoms or features related to AD pathology and may hold prognostic information [6]. Quantitative light reflex pupillometry (qLRP) is a digital bedside biomarker, measuring the static and dynamic properties of the pupillary action in response to brief light stimuli [7] and has recently demonstrated promising diagnostic capability in AD [8]. The marker is shown to change longitudinally in patients harboring amyloid [9] but whether it holds prognostic information is not known [10].

Cross-sectional studies [9, 11–14] have demonstrated that in clinical AD, the pupillary constriction is slower and pupil size is smaller compared to healthy aged-matched controls. This has been taken as evidence that the pupillary deficits are caused in part by parasympathetic failure [14]. Key regulators of the pupillary response include the Edinger Westphal nucleus (EWn) [15] locus coeruleus (LC) [16] and the dorsal raphe nucleus [17] which are located in proximity to each other in the mesencephalon and pons. These nuclei all show neuronal changes related to AD pathology, and specific brain stem affection in Alzheimer’s disease could be the cause of an altered pupillary response [18–21]. There has been an increasing focus on the role of pathology in the LC in Alzheimer’s disease as a predilection site for neurofibrillary tangles predating cortical deposition [21–23]. Moreover, significant associations between LC changes and cognitive decline have been found in AD [24] Elevated metabolism of the locus coeruleus is associated with resilience to cognitive decline [25] indicating that pupillary function may be used to probe this aspect. In addition, studies that target the pupil dilation response under cognitive paradigms have also shown an association with amnestic mild cognitive impairment and polygenic risk scores for AD [26, 27]. Peinkhofer et al. [28] have reviewed the cortical modulation of pupillary function and underscored the influence of several cortical regions, such as the insular cortex, frontal eye field and the prefrontal cortex. McDougal & Gamlin [15] provide an extensive review on the autonomic control of the eye, highlighting the pivotal role of the parasympathetic pathway in regulating the pupil. A review by Chougule et al. [14] likewise posited that parasympathetic dysfunction was the main explanation for the “sluggish” constriction of the pupil observed in several studies in patients with AD. Together, this makes an interesting case for exploring the pupillary response as a prognostic biomarker in Alzheimer’s disease. Studies investigating pupillometry in cohorts of early AD, representing a population potentially eligible for anti-amyloid treatment, are essentially missing from the literature and represent a current gap in knowledge. In this regard, both baseline measurement and short-term (months) dynamic changes in the pupillary response could be indicative of future decline, with the latter perhaps holding more information related to the disease course.

In this exploratory, prospective longitudinal study we aimed to investigate the prognostic value of baseline and short-term dynamic changes detected by quantitative light reflex pupillometry in a well-characterized cohort of early AD patients evaluated in a tertiary university memory clinic setting. We hypothesized that light reflex pupillometry is prognostic in early AD and that short-term dynamic pupillary changes can predict clinically evaluated progression within the first year after diagnosis and is predictive of progression of neurodegenerative changes as observed on [^18^F]-fluorodeoxyglucose position emission tomography ([^18^F]FDG-PET). As this was an exploratory study, our results can only be interpreted as hypothesis-generating.

## Methods and participants

### Design

Prospective, longitudinal cohort study.

### Participants

We recruited patients from a tertiary university memory clinic from January 2022 to June 2023. Diagnostic assessment at our center is comprehensive and includes examination by a physician and nurse. All patients undergo cognitive testing with two routine screening tests (Mini-Mental State Examination and Addenbrooke’s Cognitive Examination), a structural scan (computed tomography or magnetic resonance imaging), and routine blood sampling (e.g., vitamin B_12_ status). State-of-the-art neuroimaging techniques to ascertain various aspects of neurodegenerative pathology (e.g., dopamine agonist tracer scans, amyloid PET), cerebrospinal fluid biomarker analysis, genetic counseling, and neuropsychological testing, are used if deemed necessary. Patients are diagnosed following a multi-disciplinary (neurology, geriatrics, psychiatry, neuropsychology, nursing) consensus conference. The inclusion criteria were: (1) a diagnosis of MCI [29] or mild-moderate dementia due to AD [30] with and without confirmation of amyloid positivity, (2) a caregiver willing to participate as an informant, (3) a Mini Mental-State Examination total score higher than 19, (4) a baseline diagnostic hybrid brain [^18^F]FDG-PET/magnetic resonance imaging (MRI) or [^18^F]FDG-PET/computed tomography (CT), and (5) ability to cooperate to the investigations. The exclusion criteria were: (1) Current alcohol or substance abuse, (2) other major neurologic or psychiatric disorder, or (3) active participation in drug trials. This study was part of a main study (TRACK-AD) that investigated blood-based biomarkers and digital biomarkers (actigraphy and pupillometry). The TRACK-AD study was registered at clinicaltrials.gov (Clinical Trial No.: NCT05175664, registration date: 4th of January 2022). The main study was designed to both investigate prognostic and disease-tracking markers in early AD. We followed the tenets of the 1975 Helsinki Declaration.

### Procedure

Patients were included at the time of their diagnosis and followed for 18–24 months with scheduled visits at baseline, 3 months, 12 months, and 18/24 months (see study flow chart and overview in Supplementary Figs. 1 and 2). At baseline and follow-up visits, a clinical evaluation, pupillometry, actigraphy, blood sampling and cognitive testing as well as Clinical Dementia Rating (CDR) were performed (see Supplementary Material for study overview). As a screening procedure, all patients underwent ophthalmoscopy to check for obscurities of the lens and vitreous body before inclusion in the present study. The disease severity was established at each visit and classified as MCI due to AD, or mild, moderate or severe dementia according to the NIA-AA criteria for MCI due to AD [29] and the International Statistical Classification of Diseases and Related Health Problems 10th revision criteria for dementia [31]. For the present study, we focused on the immediate prognostic information of light reflex pupillometry (within one-year follow-up), in line with a previous study on neurodegenerative biomarkers [32]. All study procedures were performed on the same day, except follow-up neuroimaging which was performed approx. two weeks prior to the 1-year visit.

### Demographic data

Data on demographic variables (e.g., age, sex, educational level), and relevant comorbidities such as eye disease were extracted from the electronic health record. We also obtained a history from the caregivers and participants of any relevant eye disease and inspected medical files for prescription medication for eye-related disorders. Medication that could influence the pupil was noted according to Kelbsch et al. [33] and included both eye medication (e.g., glaucoma, allergy, other) and systemic treatment (e.g., opioids, dopaminergic agents, cholinergic medication). Eleven patients were excluded post-hoc in the present study due to severe eye disease (e.g., severe glaucoma, cataract or retinopathy) due to interference with pupillometry. The presence of severe eye disease was evaluated by the investigator (M.G.).

### Clinically evaluated progression

The primary outcome was clinically evaluated progression as rated at the 1-year follow-up visit by an experienced dementia physician. The physician determined the disease course leading up to each of the follow-up visits as either progressive or stable. The clinician was not blinded to clinical information and light reflex pupillometry nor follow-up scans, but the decision was based on the sum of clinical information (clinical impression, cognitive testing, information provided by the informant), and the rating was not necessarily carried out by the same physician at each visit. This approach was similar to that applied in a recent study investigating prognosis in a mixed memory clinic cohort [32]. The short-term dynamic pupillary changes from baseline to 3-months FU were not calculated until after the study had completed.

### Cognitive scales

Rating on the CDR scale, a semi-quantitative interview-based scale rating cognitive and functional decline [34] was carried out by an experienced rater as part of the clinical examination at baseline and at the 1-year FU visit. Only the CDR Sum-of-Boxes score (CDR-SoB) was used for analysis. The Mini-Mental State Examination (MMSE), a brief cognitive screening test [35] was administered by a nurse during the initial diagnostic work-up if this was within 4 months of the baseline visit. Otherwise, the investigator administered these tests at the baseline visit. At all subsequent visits, the investigators (M.G. and F.K.C) administered the cognitive tests and rating scales.

### [^18^F]FDG-PET

All [^18^F]FDG-PET images were obtained on clinical PET/CT or PET/MRI systems according to international standard operating procedure [36]. Baseline and follow-up scans were obtained on an identical system in 73% of cases (all Siemens PET/MRI) or comparable systems (baseline Siemens PET/CT and follow-up PET/MR) in the rest of cases, except for two cases with baseline scan performed on a GE PET/CT system and follow-up on Siemens PET/MR. Scans were assessed using statistical surface projections (Siemens Syngo.via, MI neurology, Erlangen, Germany) rated by two experienced nuclear medicine physicians (OMH, IL) in a blinded fashion with a forced decision between progressive or stable cerebral metabolism as a proxy of neurodegeneration. As such, the outcome was visually noticeable neuroimaging disease progression. Projections of baseline and follow-up scans were presented side-by-side in random order to minimize forecast bias. Scans where randomization led to reverse chronological order and were rated as progressed initially, were changed to stable after unblinding of order. If there was disagreement between raters, a consensus was reached referencing the original [^18^F]FDG-PET images fused to MRI or CT after unblinding the order.

### Quantitative light reflex pupillometry

The procedure used for qLRP has been described previously [8] where baseline visit cross-sectional qLRP data from this cohort are also reported. Briefly, a hand-held research grade pupillometer (PLR-3000, NeurOptics^®^) was used with customized settings to measure the resting pupillary diameter and pupillary light reflex (PLR). qLRP was carried out by the investigators (M.G. and F.K.C) at the baseline and subsequent visits under ambient lighting in the examination rooms of the memory clinic. The following parameters are given by the pupillometer: Resting pupil diameter (before light stimulus, mm), peak constriction pupil diameter (mm), latency (time in seconds after stimulus till constriction occurs), delta (relative percentage change between baseline and peak constriction diameter), average and maximum constriction velocity (mm/s), average dilation velocity (mm/s), and T75 (time in seconds to reach 75 of baseline value after light stimulus). A table summarizing the physiological interpretation of each parameter is included as Supplementary Table 1. The same procedure was applied at subsequent follow-up visits, ensuring similar ambient lighting within a predefined range (approx. 500–800 lx) using dimmer light switches and measurement of the light intensity near the head of the participant using the Light Meter app (using the most recent version) on an iPhone 12 or higher.

### Data quality

The method for quality checking pupillometry data has been described previously [8]. Briefly, recordings flagged by the pupillometer were visually inspected for blinking artifacts during the constriction phase and discarded if present. In the present study, a total of 156 recordings (11% of all recordings) were flagged by the pupillometer and 99 recordings (7% of all recordings) were discarded after visual inspection.

### Statistical analysis

All statistical analysis was carried out using R (ver. 4.4.2) with the integrated development environment RStudio (ver. 2024.09.1) [37].

Quantitative variables are presented by their means and standard deviations or median and range as appropriate. Normality was assessed by qq-plots and equality of variance was assessed with an equal variance test. Linear models were fitted for CDR SoB and MMSE as the dependent variables and each of the eight qLRP parameter (both entered as baseline measurements and short-term dynamic changes) as the independent variables in both crude and adjusted models. We used the mean of the first three recordings as the unit of analysis for all qLRP metrics. The short-term dynamic change was calculated by subtracting the baseline measurement from the 3-month measurement. The adjustment differed for models using baseline measurements (baseline models) and short-term dynamic changes (dynamic change models) as predictors. Baseline models were adjusted for age, sex, and resting pupillary diameter. Dynamic change models were additionally adjusted for the baseline measurement of the included qLRP parameter to reflect relative short-term dynamic changes. Models that survived the above-mentioned adjustment were further adjusted for medication that could affect the pupil and the presence of mild eye disease, separately. The CDR-SoB was log-transformed using the natural logarithm to alleviate violation of the linearity assumption. As such, coefficients from these models should interpreted by 1 unit increase in the predictor variable corresponding to the percentage change in the CDR SoB score. Logistic regression models were fitted with clinically evaluated progression and visual [^18^F]FDG-PET progression as outcomes. The outcome was coded for 1-year clinically evaluated progression as progression occurring at either the 3-month or 1-year FU. Prediction error for the logistic regression models were evaluated by the area under the receiver operating characteristics curve (AUC) with an associated 95% confidence interval using the DeLong method. To assess robustness of these estimates on unseen data, a five-fold, 10 times cross-validation was performed using the *caret* package in R for the best models as indicated by lowest Akaike information criterion (AIC) value identified using the *MuMIn* package in R, which tests all combinations of univariable models. Cut points were found by optimizing the Youden index and the sensitivity and specificity were reported with an associated 95% CI calculated with bootstrap estimation using the *cutpointr* package in R. Relevant assumptions (linearity, normally distributed residuals, and absence of significant heteroscedasticity) were met for all linear and logistic regression models. Partial regression plots were constructed for significant predictors in the linear models showing the regression line with the associated 95% prediction interval, keeping all other variables at the mean value and categorical variables to reference values. A *p*-value < 0.05 was considered significant and only two-tailed tests were used. Due to the exploratory nature of the study, no adjustments for multiple comparisons were made, and our results are only interpreted as hypothesis-generating.

## Results

**Fig. 1:**
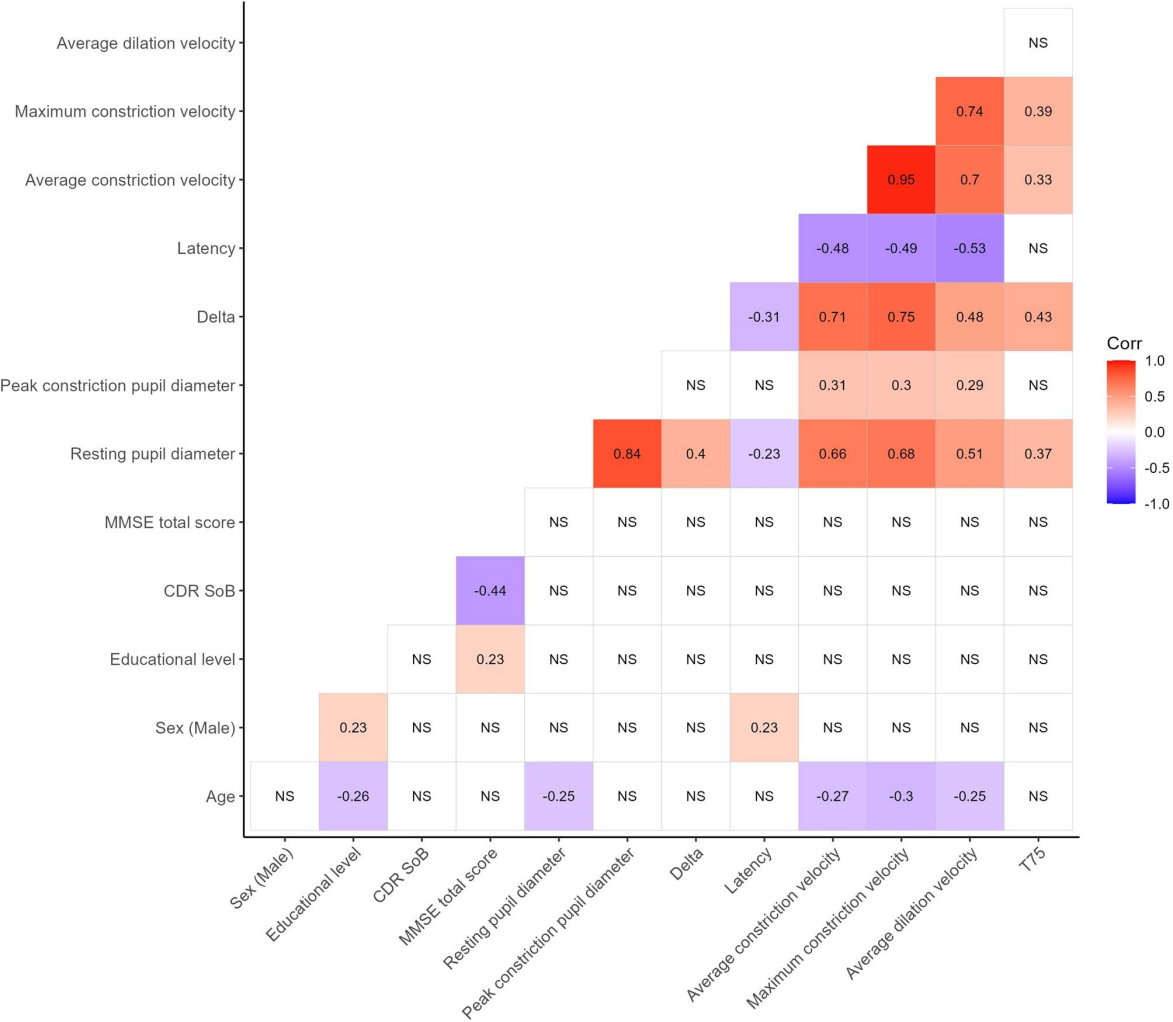
Correlogram showing Spearman’s ρ for significant correlations, NS = not significant

**Fig. 2:**
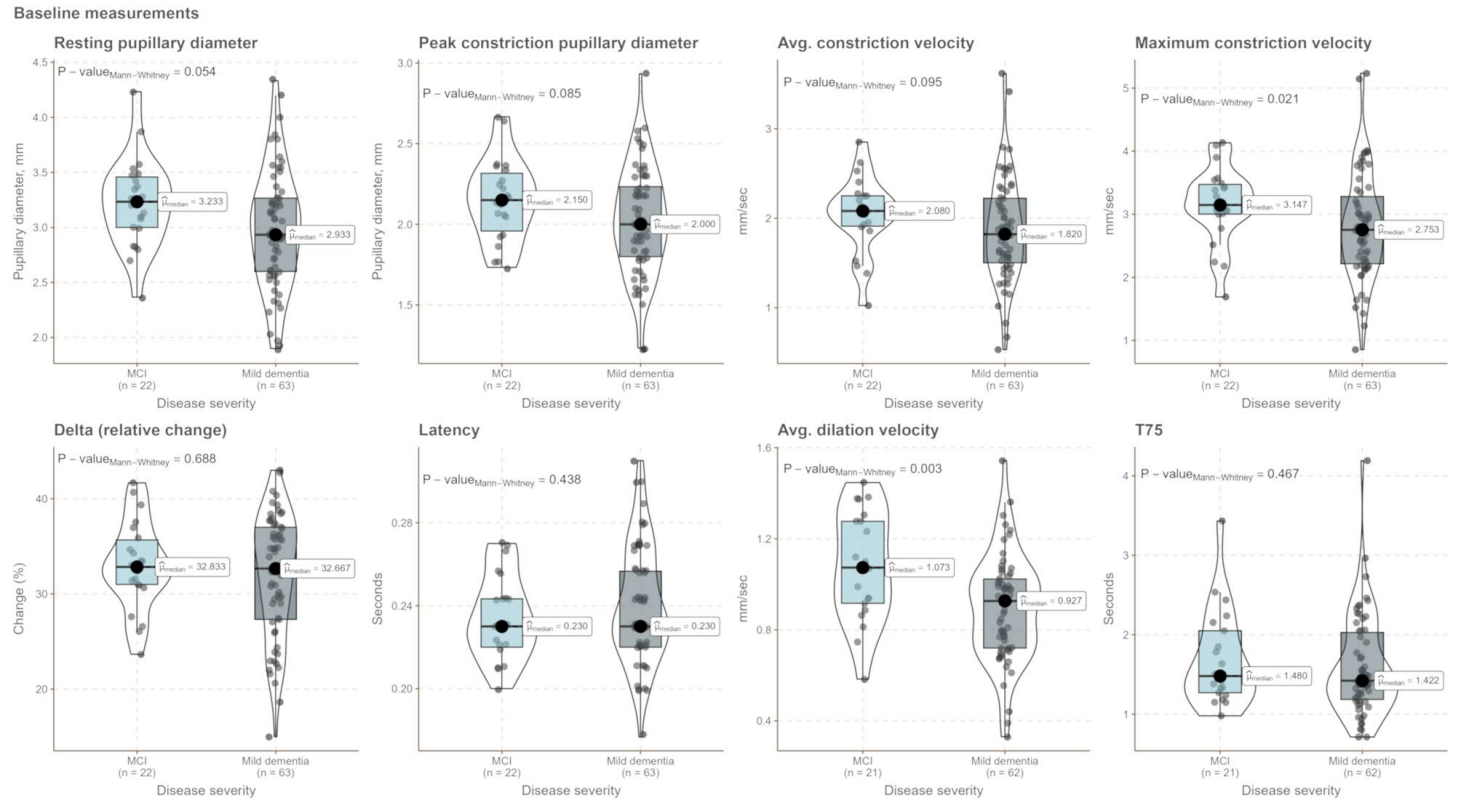
qLRP parameters measured at baseline stratified according to disease severity at baseline. One patient had moderate dementia at baseline (not shown in plot). MCI: mild cognitive impairment

**Fig. 3:**
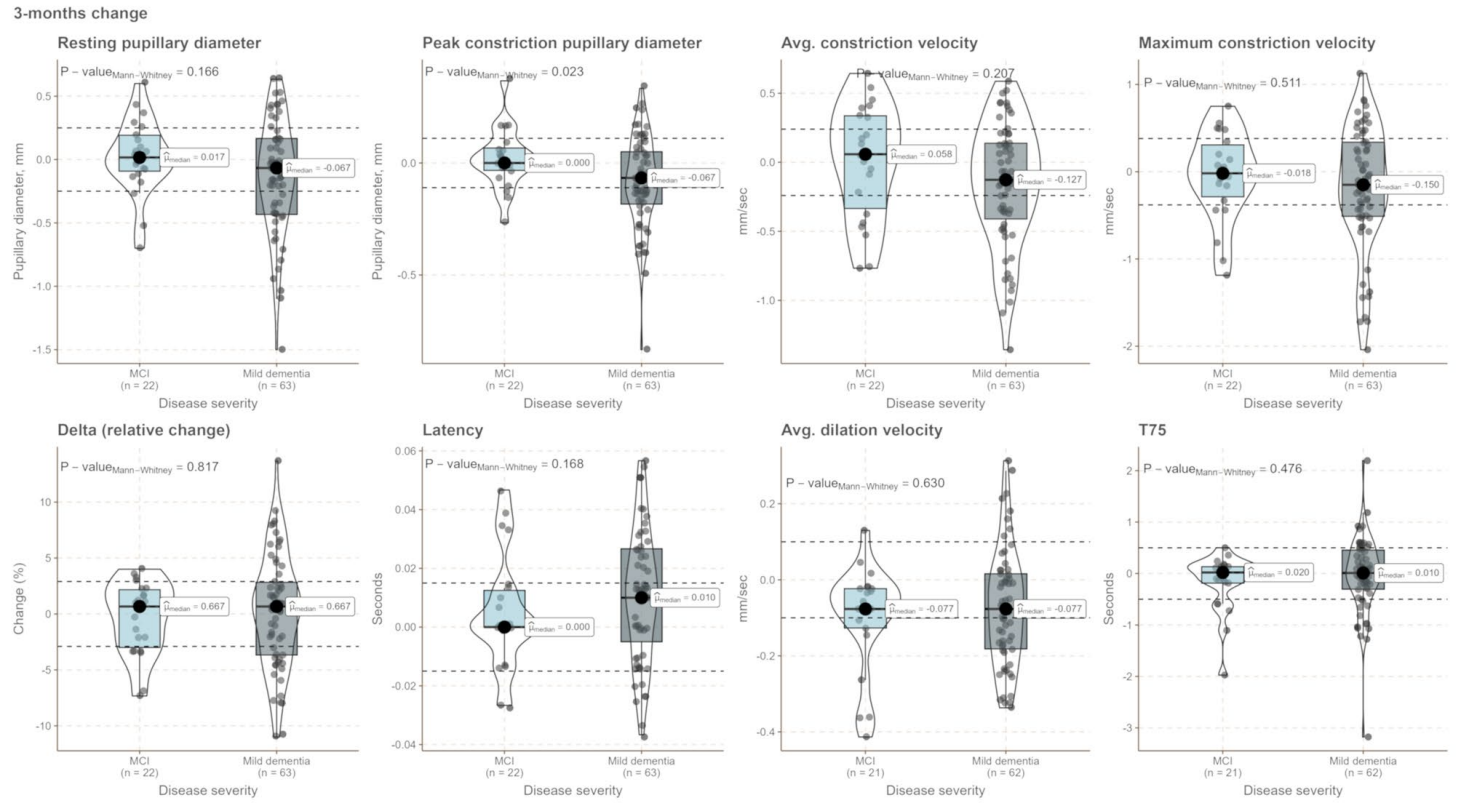
Short-term dynamic changes in qLRP parameters stratified according to disease severity. As only one patient had moderate dementia at baseline, this patient was omitted from the plot. MCI = Mild cognitive impairment

In Table 1, patient characteristics for the included 86 patients are shown stratified according to clinically evaluated progression status at the 1-year follow-up visit. Patients were followed for a median of 1.66 years (inter-quartile range: 1.479–1.96 years). During the study period, there was a loss to follow-up of 17 patients and one patient changed diagnosis at the 3-month visit and was excluded from analysis (see study flow chart in Supplementary Fig. 1). Patients who progressed were significantly older (*p* = 0.021). There was an equal distribution of the sexes and groups were balanced at baseline on the MMSE and CDR-SoB. No differences were observed for mild, chronic eye disease or medication that could influence pupillary function. There was an equal proportion of patients receiving treatment with a cholinergic drug at the 3-month visit. qLRP parameters were highly correlated, and baseline pupillary diameter at baseline was significantly correlated with all other qLRP variables (Fig. 1). When stratifying patients cross-sectionally at baseline according to disease severity, there was a tendency towards a slowing of the pupillary response (*p* = 0.021 for baseline maximum constriction velocity) with increasing disease severity. There was an increased variability, as appreciated visually, of the pupillary response in the mild dementia group (Figs. 2 and 3).

**Table 1:** Cohort characteristics at the baseline visit stratified according to progression status at 1-year follow-up

	Stable (*N* = 39)	Progression (*N* = 47)	Total (*N* = 86)	*p*-value
**Age (years)**				**0.021** ^1^
Mean (SD)	73.8 (7.4)	77.3 (6.4)	75.7 (7)	
Range	53.3–89.9	63.7–88.2	53.3–89.9	
**Sex**				0.709^2^
Male	19 (48.7%)	21 (44.7%)	40 (46.5%)	
Female	20 (51.3%)	26 (55.3%)	46 (53.5%)	
**Educational level (years)**				0.315^3^
Mean (SD)	11.2 (3.4)	12.2 (4)	11.7 (3.7)	
Range	7–20	7–20	7–20	
**Disease severity**				0.542^2^
MCI	10 (25.6%)	12 (25.5%)	22 (25.6%)	
Mild	28 (71.8%)	35 (74.5%)	63 (73.3%)	
Moderate	1 (2.6%)	0 (0.0%)	1 (1.2%)	
**MMSE total score**				0.758^1^
Mean (SD)	25.4 (2.6)	25.6 (2.4)	25.5 (2.5)	
Range	20–30	21–30	20–30	
**Clinical Dementia Rating Sum-of-Boxes score**				0.157^3^
Mean (SD)	2.5 (1.1)	3 (1.6)	2.8 (1.4)	
Range	0.5–5	1–9.5	0.5–9.5	
**Mild**,** chronic eye disease**				0.237^2^
No	28 (71.8%)	28 (59.6%)	56 (65.1%)	
Yes	11 (28.2%)	19 (40.4%)	30 (34.9%)	
**Medication with pupil effect**				0.133^4^
No	33 (84.6%)	45 (95.7%)	78 (90.7%)	
Yes	6 (15.4%)	2 (4.3%)	8 (9.3%)	
**AChEI treatment at 3-month FU**				0.265^2^
No	17 (43.6%)	15 (31.9%)	32 (37.2%)	
Yes	22 (56.4%)	32 (68.1%)	54 (62.8%)	
**Visual progression on PET**				
N missing	6	12		**0.029** ^2^
No	21 (63.6%)	13 (37.1%)	34 (50%)	
Yes	12 (36.4%)	22 (62.9%)	34 (50%)	

^1^Unpaired T-test (Welch), ^2^Pearson’s χ^2^-test, ^3^Wilcoxon rank sum test, ^4^Fisher’s exact test. MMSE: Mini Mental-State Examination, ACE: Addenbrooke’s Cognitive Examination, AChEI: acetylcholinesterase inhibitor. **Bold** indicates significance

### Prognostic models – clinically evaluated progression (1-year)

**Fig. 4:**
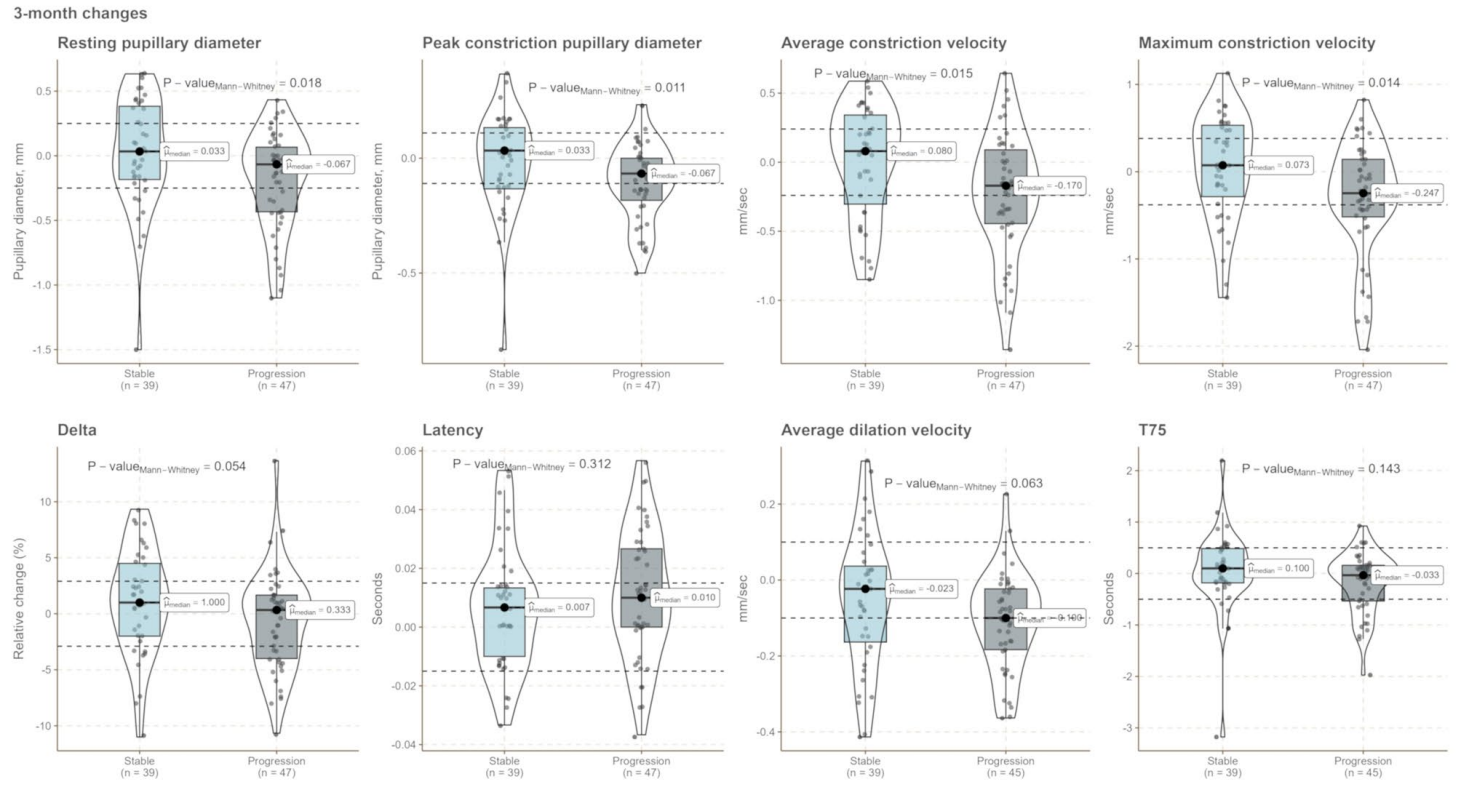
Short-term dynamic changes in pupillary parameters from baseline-3-month FU. *P*-values reflect the significance of differences between Progression and Stable groups. The dashed line represents the short-term variability that could be considered due to random fluctuations as based on a previous study [43]

**Fig. 5:**
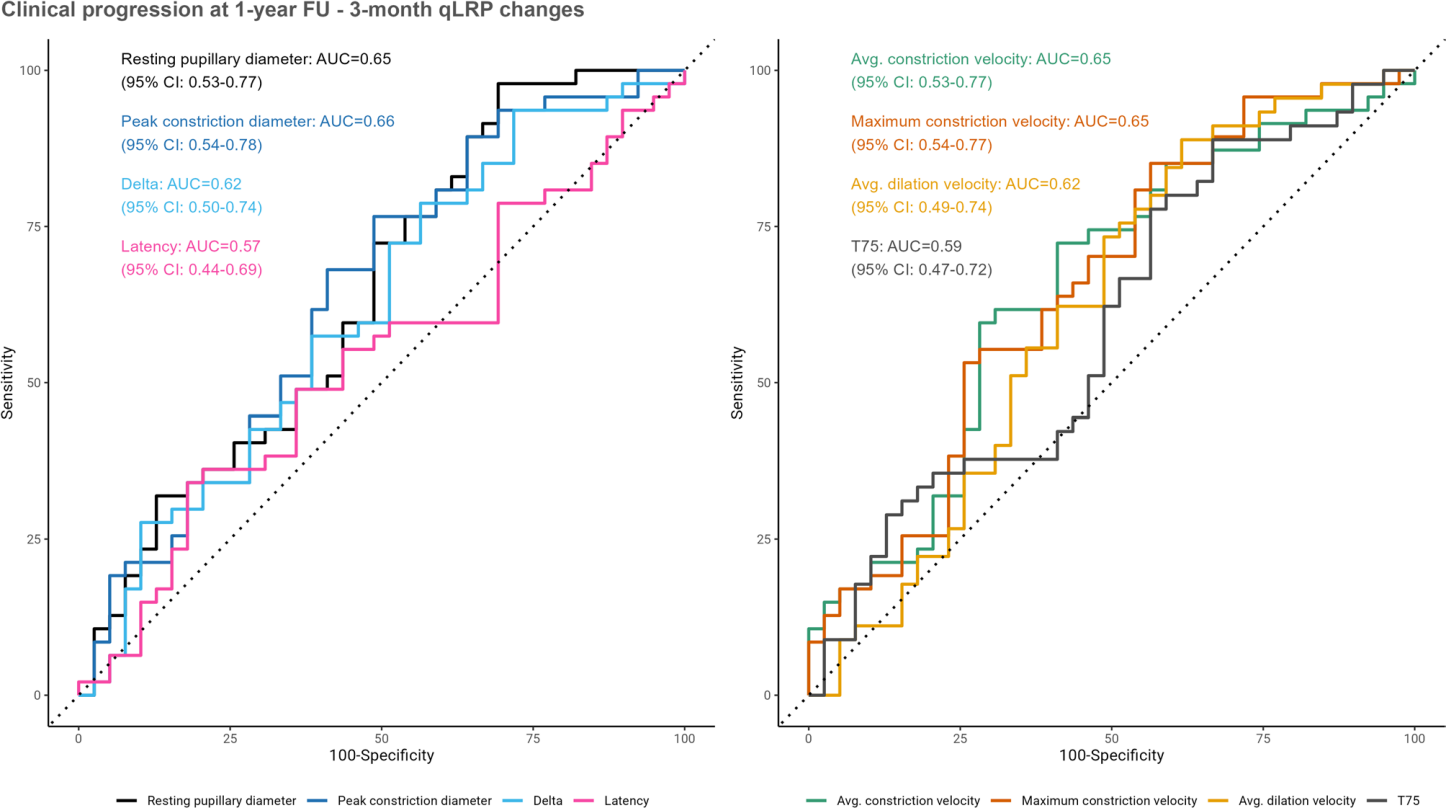
Model performance of individual short-term dynamic qLRP changes shown as receiver operating characteristics (ROC) curves for predicting clinically evaluated progression. The 95% confidence interval (95% CI) is calculated with the DeLong method

For short-term dynamic changes in qLRP parameters, several parameters (resting and peak constriction pupillary diameter, average and maximum constriction velocity) showed a statistically significant difference between stable and progressed patients (Fig. 4). Progressors had smaller resting pupil sizes (*p* = 0.018) and exhibited slower average constriction velocity (*p* = 0.015) with a trend towards smaller delta values (*p* = 0.054) (Fig. 4), whereas baseline qLRP parameters were not significantly different between groups (Supplementary Tables 3 and Supplementary Fig. 3). Several short-term dynamic qLRP changes, such as resting and peak constriction pupillary diameter and the velocity of constriction, were univariately associated with progression at 1-year FU and could predict this outcome above chance level (Table 2; Fig. 5). Following adjustment, only decreases in the resting pupillary diameter remained associated with clinically evaluated progression (Table 2). The multivariable models had similar AIC values (data not shown), i.e., there was no additive value by combining predictors, and therefore the best model was chosen based on the variable surviving adjustment for age and sex. Dynamic changes in the resting pupillary diameter had an associated cross-validated AUC of 0.66 (SD: 0.13), a sensitivity of 98% [95% CI: 15–100%], and a specificity of 31% [95% CI: 13–91%] for predicting clinically evaluated progression. This present study reports a 1-year progression rate of 54%, which would in turn provide a negative predictive value of 93% and a positive predictive value of 63% for predicting 1-year progression using short-term dynamic changes in the resting pupillary diameter.

**Table 2:** Logistic regression models with 1-year clinically evaluated progression (progressor vs. stable at 1-year follow-up) as the outcome and short-term dynamic changes (3-month Δ) as predictors. Odds ratios are displayed as reciprocal to indicate the estimate for a 1 unit decrease in the qLRP variables

	Univariable		Adjusted	
	Odds ratio [95% CI]	*p*-value	Odds ratio [95% CI]	*p*-value
Resting pupil diameter (3-month Δ)	3.9 [1.3, 14]	**0.022**	4.28 [1.24, 16.9]	**0.028** ^**a**^
Peak constriction pupil diameter (3-month Δ)	13.6 [1.3, 192.1]	**0.039**	3.02 [0.001, 70.6]	0.7
Delta, relative pupillary change (3-month Δ)	1.09 [0.99, 1.21]	0.078	1.03 [0.89, 1.22]	0.6
Latency (3-month Δ)	-	0.3	-	0.8
Average constriction velocity (3-month Δ)	3.62 [1.28, 11.4]	**0.020**	1.16 [0.09, 14.8]	> 0.9
Maximum constriction velocity (3-month Δ)	2.5 [1.23, 5.59]	**0.016**	2.21 [0.34, 14.5]	0.4
Average dilation velocity (3-month Δ)	12.1 [0.7, 262.6]	0.10	2.53 [0.02, 303.7]	0.7
T75 (3-month Δ)	1.64 [0.86, 3.5]	0.2	1.99 [0.67, 6.65]	0.2

Adjustment was age, sex, baseline qLRP variable and change in pupil diameter from baseline-3-months (except for in ^a^). The estimate for odds ratio and the 95% CI for latency is not shown, as it was extremely high due to little variation in the variable. **Bold** indicates significance

### Clinical dementia rating sum-of-boxes

We evaluated the association between qLRP measurements at the baseline visit as well as 3-month dynamic changes with the annualized change in the CDR-SoB. The variance of the CDR-SoB score was large (see Supplementary Fig. 4), and also larger than for MMSE (data not shown). We did not find any significant associations between qLRP and CDR-SoB (see Supplementary Tables 4 and 5). Nonetheless, the estimates were in a similar direction as those observed for clinically evaluated progression.

### Cognitive decline – MMSE

**Fig. 6:**
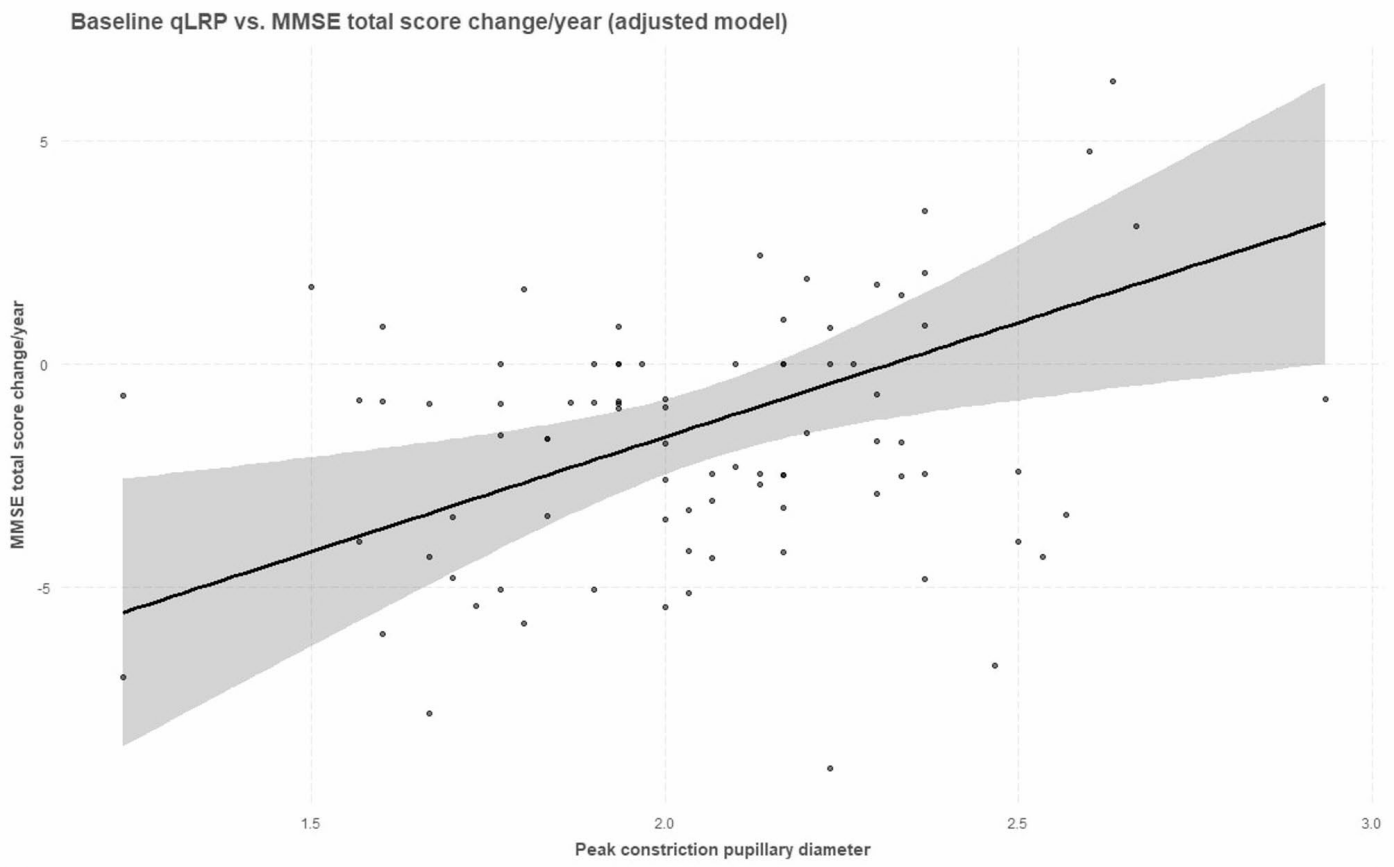
Partial regression plot showing the association between baseline peak constriction diameter and MMSE annualized Δ. The adjustment was for age, sex and resting pupillary diameter

Baseline measurements of both peak constriction diameter and the delta variable were significantly associated with cognitive decline on MMSE, also when adjusting for relevant confounders (Table 3; Fig. 6). For 3-month changes in qLRP, no statistically significant associations were found (Supplementary Table 6).

**Table 3:** Results of a linear regression model showing the association between MMSE annualized change (1-year) and qLRP variables (baseline measurements). Adjustment was for age, sex and resting pupil diameter, except for the model with this variable as the predictor

Characteristic	Univariable			Adjusted		
	Beta	95% CI	*p*-value	Beta	95% CI	*p*-value
Resting pupil diameter (mm) (baseline)	0.45	-0.67, 1.6	0.4			
Peak constriction pupil diameter (mm) (baseline)	2.0	0.18, 3.8	**0.031**	5.1	1.6, 8.6	**0.004**
Delta, relative pupillary change (percent (baseline))	-0.11	-0.21, -0.01	**0.024**	-0.16	-0.26, -0.05	**0.004**
Latency (s) (baseline)	-1.8	-24, 20	0.9			
Average constriction velocity (mm/s) (baseline)	-0.20	-1.3, 0.88	0.7			
Maximum constriction velocity (mm/s) (baseline)	-0.04	-0.77, 0.69	> 0.9			
Average dilation velocity (mm/s) (baseline)	1.8	-0.60, 4.1	0.14			
T75 (s) (baseline)	-0.53	-1.5, 0.39	0.3			

### Visual [^18^F]FDG-PET progression

**Fig. 7:**
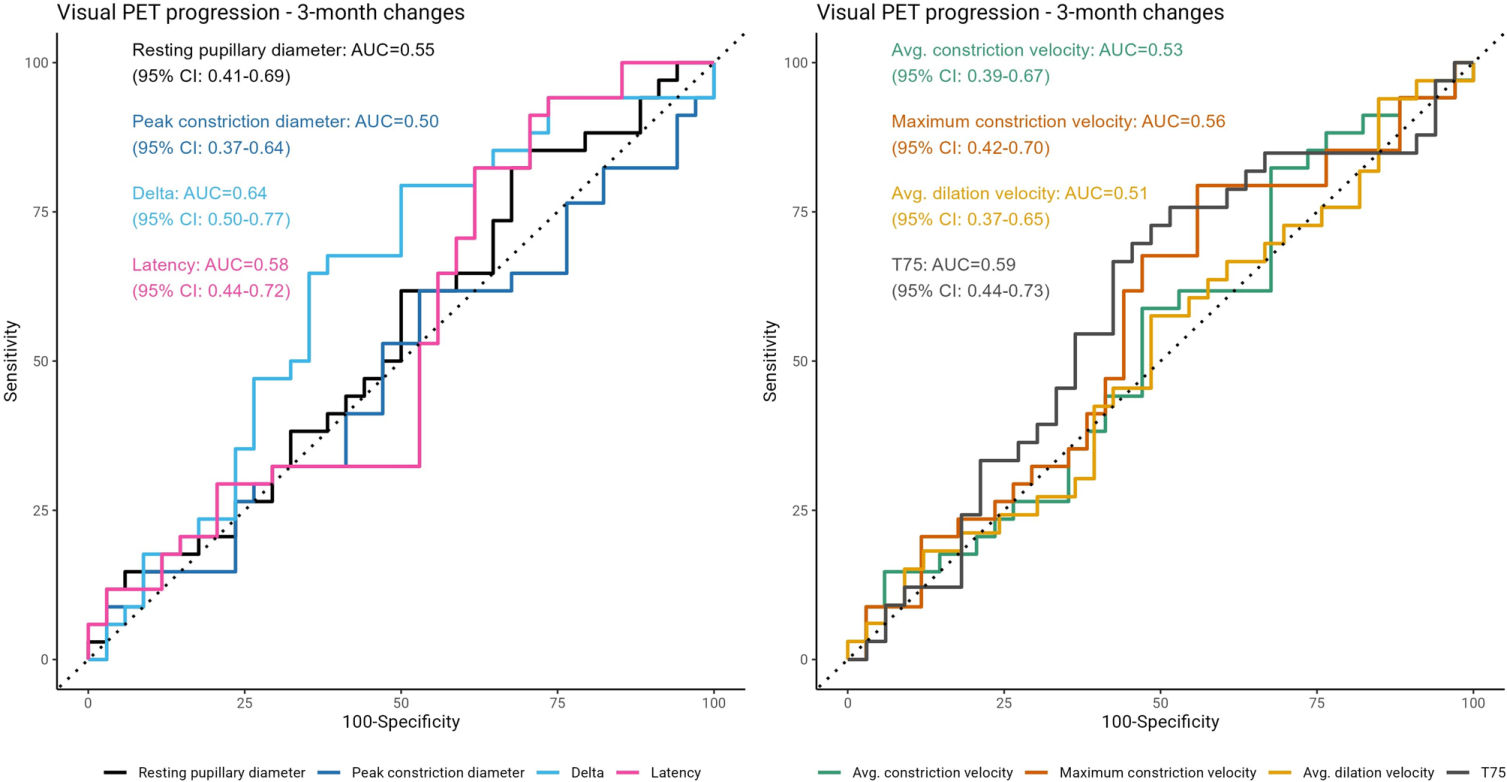
Receiver operating characteristics (ROC) curves for the prediction of visual [^18^F]FDG-PET progression for individual qLRP parameters

We evaluated the predictive value of qLRP on progression as assessed on a visual [^18^F]FDG-PET read, whereby visual changes as shown on a cortical projection image were detectable by two experienced nuclear medicine physicians. In general, the agreement between clinical and neuroimaging progression was poor, indicated by Cohen’s κ = 0.27, however there was some overlap between clinical progressors and neuroimaging progressors (Table 1). Only short-term dynamic decreases in the delta qLRP variable (relative pupillary change) were predictive of progression on [^18^F]FDG-PET (cross-validated AUC 0.64, 95% CI 0.5 − 0.07), but this variable was not significantly associated with neuroimaging progression in the logistic regression models (see Fig. 7 and Supplementary Tables 7 and 8). The associated sensitivity was 68% [95% CI: 25–100%] and the specificity was 62% [95% CI: 17–83%]. The directions of estimates for the logistic regression models were similar to clinically evaluated progression for short-term dynamic changes.

### Confounder analysis

While no statistically significant differences were present at baseline between progressors and stable patients for medication that could affect the pupil and mild eye disease, there was a numerically lower proportion of progressors receiving these types of medications. There was also a numerically higher proportion of patients with mild eye disease in the progressor group. This could lead to confounding and we therefore performed a further multivariable adjustment including these variables. The analyses showed that the association between short-term dynamic changes in the resting pupillary diameter and clinically evaluated progression could be explained by adjusting for medication that could affect the pupil but not when only adjusting for eye disease (see Supplementary Table 9). Taking medication that could affect the pupil was not however associated with either baseline (Unpaired T-test *p* = 0.697) nor short-term dynamic changes (Unpaired T-test* p* = 0.101) in the resting state pupil. The associations were unaltered and the statistical significance was intact when adjusting for these confounders in models with MMSE annualized change as the outcome (data not shown).

## Discussion

In this study we evaluated the prognostic performance of qLRP in early Alzheimer’s disease. Our results showed that short-term dynamic changes in the pupillary resting diameter were predictive of progression at 1-year follow-up using clinically evaluated progression as an outcome. This indicates the possible usefulness of qLRP as a bedside prognostic tool. This was further corroborated by associations with cognitive decline as measured with MMSE and cross-sectional differences in qLRP between disease stages, while significant associations with functional decline as measured on the CDR Sum-of-Boxes were not found, maybe due to high variance in the CDR-SoB. Short-term dynamic changes in the qLRP delta variable were predictive of progression on visual [^18^F]FDG-PET read indicating that qLRP marker may also be used to predict global neurodegenerative changes as observed with neuroimaging.

We identified the resting pupillary diameter, a general measure of arousal [38] to be predictive of clinically evaluated progression in early Alzheimer’s disease. As hinted previously, neuropathology of the brain stem in the dorsal raphe nucleus may explain why arousal and disease progression are connected in AD, as early pathology is present in the brain stem before cortical areas such as the transentorhinal cortex [20, 39]. Further, brain areas associated with the presence of apathy, a marker of rapid cognitive decline [40] are involved in arousal systems [41]. These notions align with our main finding of the resting pupillary diameter as a marker of future progression. This is to our knowledge the first study to investigate qLRP as a bedside prognostic marker, and as such represents the first proof-of-concept for using pupillometry to gauge prognostic aspects in AD. Previous studies [9, 42] have generally identified a slowing of the contracting phase after a light stimulus in AD cross-sectionally [14] although few studies adjusted for resting pupillary diameter and age of participants. In our study, constriction velocity was not associated with clinically evaluated progression after adjustment for resting pupillary diameter, which seems appropriate, as we have previously shown the resting pupillary diameter to be strongly correlated with all pupillary metrics [8, 43] indicating a hierarchical physiological system. Our adjustment for age was justified by the significant baseline correlations found between this variable and several qLRP parameters, which has also been demonstrated by others [44]. As the progressors were older on average, this was an important confounder to consider and indeed most estimates diminished following adjustment for this factor.

Generally, the changes observed on the group level were small and some of the patients in the stable group showed short-term increases in pupillary diameter, which we cannot explain from a physiological perspective. This could indicate that the marker exhibits more variability than previously shown [43] and should warrant some caution in the interpretation of our results. We found a high negative predictive value for short-term dynamic changes in the resting pupil diameter, providing the ability to reassure patients of stable disease course within a year. However, the positive predictive value was only slightly higher than the base rate of progression, indicating that it should not be used for indicating a high likelihood of progression. Indeed, the AUC was only moderate and the standard deviation for the cross-validated AUC was high, indicating low robustness of our findings. This could be owing to the either a small effect size or the moderate number of progressors, that was borderline for the conservative rule of thumb of 10 events per variable [45]. This was likewise reflected in the confounder analysis where adjustment for medication that could affect the pupil overturned the significance of the association, diminishing the belief of a true association. We also observed a tendency for patients at later disease stages to exhibit a higher variability of the marker at baseline, indicating that the constriction velocity may become unstable as the disease progresses. This suggests that qLRP may best reflect underlying disease progression in the very early (MCI) stages of AD, although we did not investigate this aspect due to our limited sample size. Future studies should investigate whether the volatility of qLRP measurements could serve as a stage marker for AD.

We showed that the qLRP variable delta was predictive of visual progression on PET, while we could not replicate the finding for resting pupillary diameter as shown for clinically evaluated progression. This suggests that the outcomes were not completely congruent, and indeed the agreement between clinical and neuroimaging progression in this study was poor. The more global patterns observed on visual [^18^F]FDG-PET reads may not reflect subtle changes in strategic areas important for cognition and independent living and some patients show little impact on ADL function even when larger areas show cumulative hypometabolism, especially in earlier disease stages [46]. This could be mitigated by applying voxel-wise statistics, which we intend to do in future analyses.

While we observed significant differences in dynamic changes of qLRP metrics and disease stage, we could not establish an association with cognition at the baseline visit, which has previously been shown [42]. This could be explained by the early disease stage, as reflected by the higher mean MMSE, resulting in a small variation in the variable, as MMSE exhibits a ceiling effect [47]. This correlation should preferably be examined in a longitudinal set-up where changes in one variable could be correlated with changes in the other. This would increase power, which is lower for cross-sectional comparisons [48].

Our cohort resembles those of previous studies regarding the rate of progression (approx. 2 points decline per year on the CDR SoB) [49] and we confirm the larger variance usually observed in the CDR score rendering it more suitable for larger cohort studies [2]. We believe that the null findings for this outcome may be owing to the large variation which was confirmed by evaluating the variance of the CDR-SoB, which increased with each visit, more so than for MMSE. It seems that, as the disease progresses, the CDR takes on a larger variability, as we also observed for qLRP. The increasing variability of the CDR and the limited capability of MMSE to gauge progression [50] makes the case that investigating clinically evaluated progression as evaluated by a comprehensive clinical evaluation is a valid construct that could supplement these investigations. This is not to say that clinically evaluated progression is without faults, as it could be driven by individual factors related to the examining clinician, which we did not characterize in the present study. Looking at several progression outcomes and thereby seeking to validate findings across outcomes could prove a useful method, as we also apply in this study. However, while we validate some findings across the clinical measures, for example regarding the delta variable, other findings could not be replicated limiting the validity of our results. While we do provide adequate adjustment for confounders, there is also a high likelihood of type I error due to the multiple outcomes and our choice of investigating all eight qLRP parameters. Choosing to investigate all eight qLRP metrics was a deliberate choice as no other studies have looked at these variables in a prognostic context. Thus, it naturally follows that our results can only be interpreted as hypothesis-generating.

A strength of our study is the well-characterized, prospective cohort with generalizable inclusion criteria. Also, as the cohort was at a very early stage of disease our results become more applicable in the future of disease-modifying treatment, where the focus is on early initiation to prevent decline. In addition, we provide cross-validated estimates of our prediction models to evaluate robustness and adjust models for relevant confounders such as mild eye disease and medication with a pupillary effect. Last, using a hand-held, clinically validated, and easily applicable tool, our results could be directly translated to clinical practice, however, validation studies and a better understanding of the underlying mechanism are needed, which could be gained through quantitative neuroimaging studies.

We acknowledge that our study is not without limitations. First, we experienced a small, but non-negligible, drop-out, which may have skewed our results and limited power to draw firm conclusions. Second, a complete ophthalmological examination beyond ophthalmoscopy was not done and this may have led to some patients not having relevant eye pathology reported. Although we adjust for mild eye disease, this could have influenced our results. We await the ongoing efforts to evaluate the influence of ophthalmological comorbidities on pupillary responses in persons with dementia [51]. Third, we did not seek to validate our results in an independent cohort, however, using cross-validation, we could provide unbiased estimates of predictive power. Fourth, as we tested multiple outcomes, there may be a higher chance of spurious findings, which is why our results should be replicated by others in larger cohorts. Fifth, there is a possibility of confirmation bias stemming from the examiner not being blinded to pupillary read-outs, however, aggregated mean measures and short-term dynamic changes were not calculated until after study completion to limit this bias. Sixth, as showed with cross-validated analyses our prediction was not robust to unseen data, probably due to a moderate sample size and a high variation in the parameters in question, which limits the external validity of our findings.

In conclusion, we demonstrated the prognostic potential of qLRP as a bedside digital biomarker in a well-characterized cohort with adequate follow-up. We demonstrated that the most promising marker for predicting clinically evaluated progression is short-term dynamic changes in the resting pupillary diameter, indicating that decreases in arousal levels could be used to predict clinically evaluated progression in early Alzheimer’s disease, however the marker was not robust to confounder analysis, and some of the effects may have been driven by slight differences in the proportion of participants taking medication that could influence the pupil. Nonetheless, qLRP seems promising in providing a bedside measure that could be used, along with future prognostic markers, to inform patients and possibly guide treatment selection in Alzheimer’s disease. Future studies should evaluate pupillometry in larger cohorts along with quantitative neuroimaging and clinical measures and with adequate adjustment for confounders as well as limit comparisons to corroborate and validate our findings before clinical implementation can be envisioned.

## Electronic supplementary material

Below is the link to the electronic supplementary material.

Supplementary Material 1

## Data Availability

The dataset generated in the present study can not be shared publicly due to the Danish Data Protection Law. It is available upon reasonable request from the corresponding author.

## Code availability

The code used to generate the results of this paper is available at https://www.github.com/Matgram/progqLRP.
