# Supplementary material for: Prognostic value of light reflex pupillometry in Alzheimer’s disease – a longitudinal cohort study
Source: Alzheimers Res Ther. 2025 Jul 11;17:152. doi: 10.1186/s13195-025-01794-8 (PMC12247382; doi:10.1186/s13195-025-01794-8)
Supplement: Supplementary file 1 — Supplementary Material 1 [file 13195_2025_1794_MOESM1_ESM.docx]

Supplementary Material

Related to *Prognostic value of light reflex pupillometry in Alzheimer’s disease – a longitudinal cohort study*

# Supplementary Figures

**Supplementary Figure 1.** Flow chart for inclusion and follow-up.


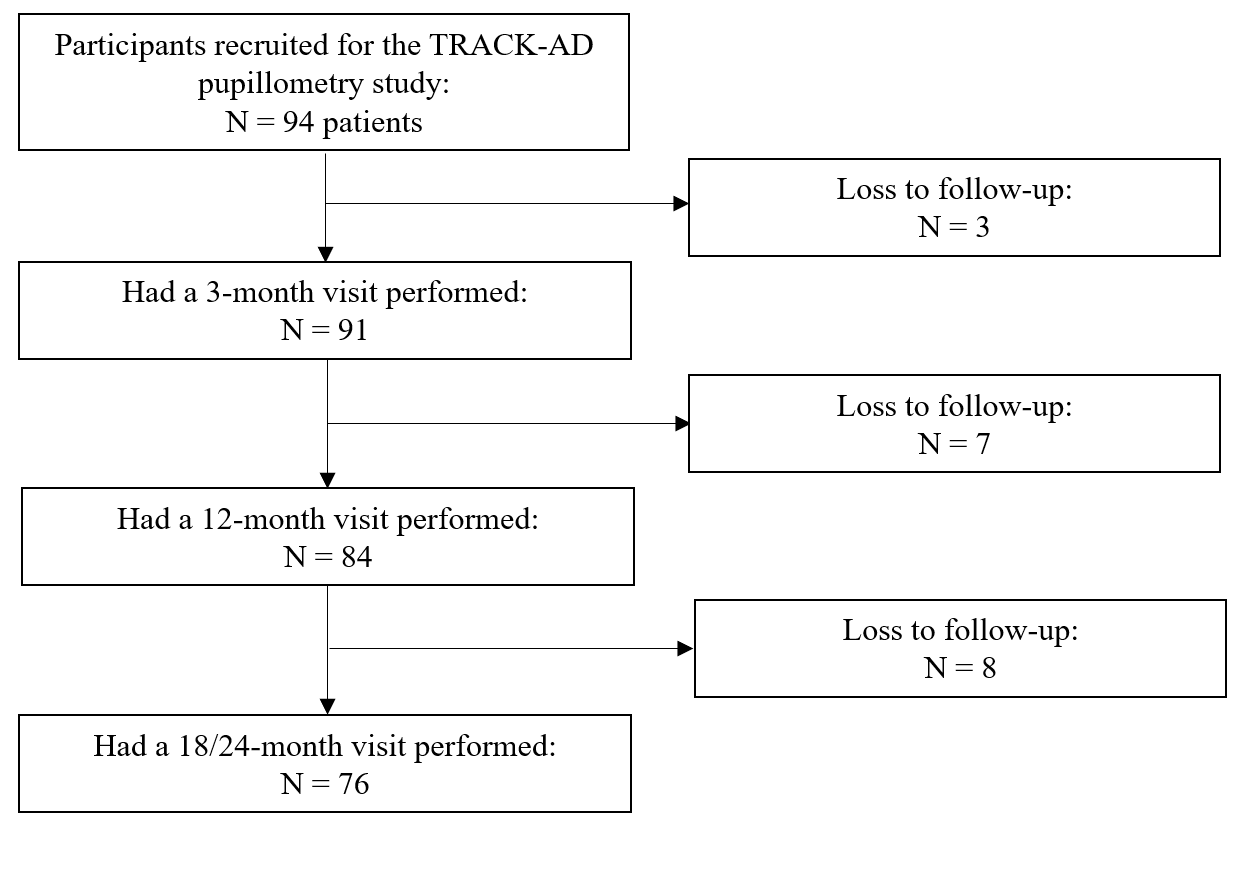


**Supplementary Figure 2.** Study overview


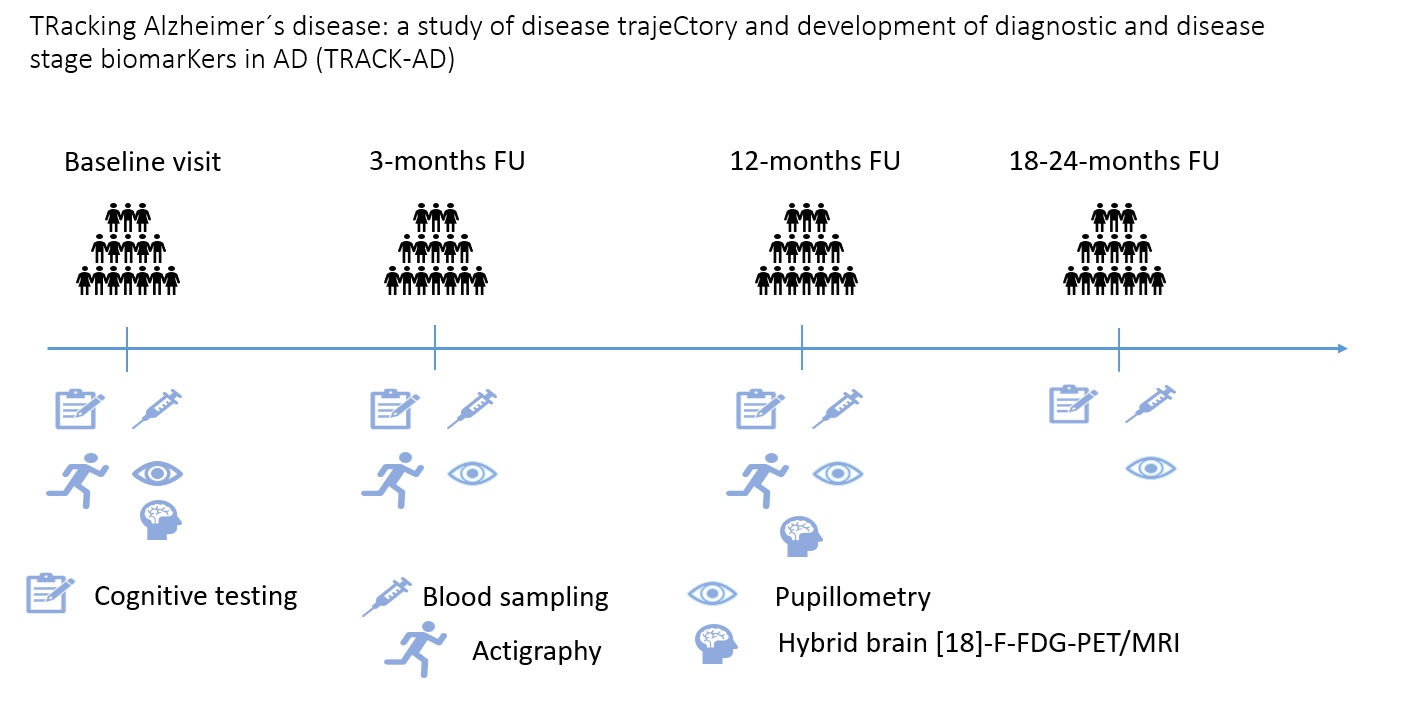


**Supplementary Figure 3.** Baseline visit measurements of qLRP and progression status at the 1-year follow-up visit.


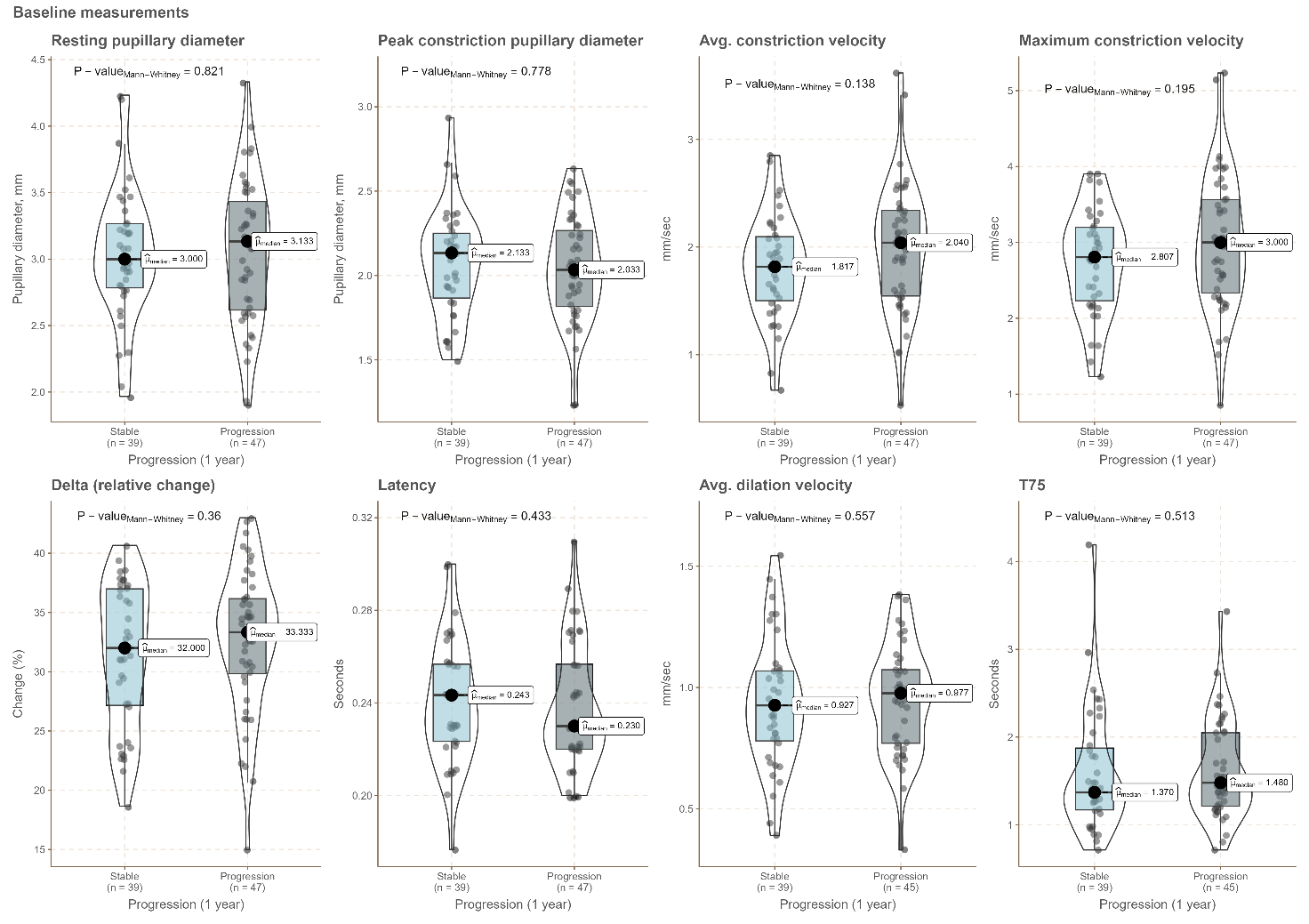


**Supplementary Figure 4**. Mean and standard deviation for the Clinical Dementia Rating Sum-of-Boxes total score for each visit.


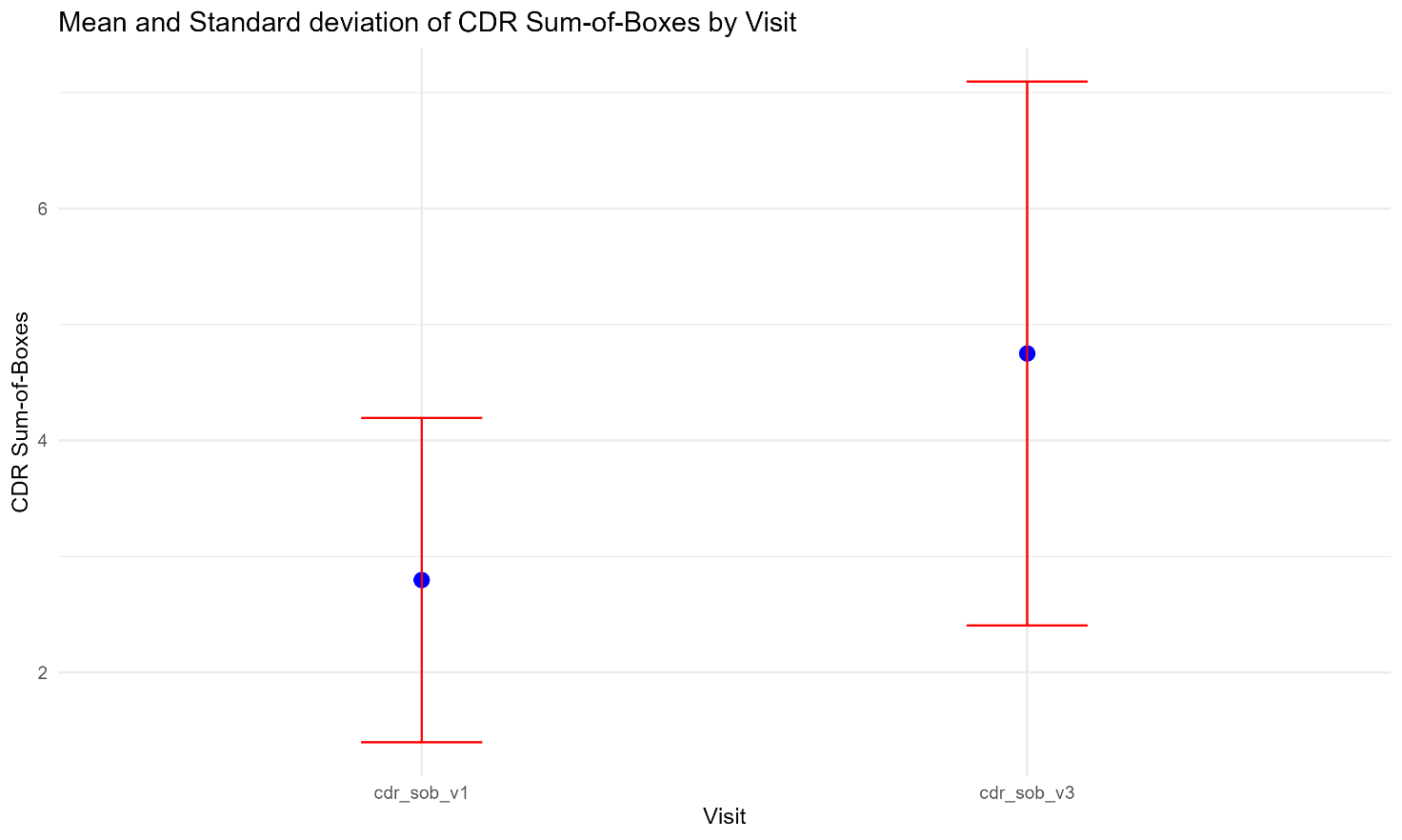


## Supplementary Tables

**Supplementary Table 1. qLRP metrics and a non-exhaustive list of the physiological interpretation of each marker as well brain areas associated with the marker.**

| **Light reflex metric** | **Physiological interpretation and brain areas associated with the marker** |
| --- | --- |
| ***Resting pupillary diameter*** | **Indicator of autonomic balance**^1^**, cognitive strain^2,3^ and arousal^4^. Brain areas: LC^3^, DRn^5^, EWn^6–8^, hypothalamus, insula, frontal eye field, colliculus superior.** |
| ***Peak constriction diameter*** | **Should be interpreted together with resting pupillary diameter, resulting in the delta value (see below). Brain areas: EWn, pretectal olivary nucleus** |
| ***Delta, relative pupillary change*** | **Reflects overall responsiveness of the pupil. Can be modulated by the frontal eye field during saccadic eye movements. Brain areas: EWn, frontal eye field (Brodmann area 8), ciliary ganglion** |
| ***Latency*** | **Represents overall nerve conductivity of the involved reflex arc, as delays could indicate demyelination^9^. Brain areas: optic nerve, chiasm and tract, pretectal brain area.** |
| ***Constriction velocity*** | **Indicator of EWn function, as the constriction mainly arises, when the neurons of this nucleus fires^1^. Brain areas: EWn, ciliary ganglion** |
| ***Maximum constriction velocity*** | **As for *constriction velocity*, but could indicate synchronicity in firing, i.e. robustness, of the EWn.** |
| ***Average dilation velocity*** | **Indicator of sympathetic recovery dynamics^1,10^.**  **Bran areas: Hypothalamus, sympathetic spinal pathways, superior cervical ganglion.** |
| ***T75*** | **Same as for average dilation velocity, although a less reliable metric according to^11^.** |

Abbreviations: EWn: Edinger-Westphal nucleus, LC: Locus coeruleus, DRn: Dorsal raphe nucleus.

**Supplementary Table 2.** Cohort characteristics at 1-year follow-up.

|  | Decline (N=47) | Stable (N=39) | Total (N=86) | p value |
| --- | --- | --- | --- | --- |
| **Disease severity** |  |  |  | < 0.001^1^ |
| N missing | 1 | 0 | 1 |  |
| MCI | 1 (2.2%) | 10 (25.6%) | 11 (12.9%) |  |
| Mild | 18 (39.1%) | 28 (71.8%) | 46 (54.1%) |  |
| Moderate | 26 (56.5%) | 1 (2.6%) | 27 (31.8%) |  |
| Severe | 1 (2.2%) | 0 (0.0%) | 1 (1.2%) |  |
| **MMSE total score** |  |  |  | < 0.001^2^ |
| N missing | 1 | 0 | 1 |  |
| Mean (SD) | 22.261 (3.242) | 24.718 (3.379) | 23.388 (3.509) |  |
| Range | 16.000 - 30.000 | 17.000 - 30.000 | 16.000 - 30.000 |  |
| **CDR Sum-of-Boxes** |  |  |  | < 0.001^3^ |
| N missing | 1 | 1 | 2 |  |
| Mean (SD) | 5.891 (2.397) | 3.368 (1.329) | 4.750 (2.345) |  |
| Range | 2.000 - 11.000 | 0.000 - 8.000 | 0.000 - 11.000 |  |

1. Pearson’s Chi-squared test
2. Linear Model ANOVA
3. Kruskal-Wallis rank sum test

**Clinically evaluated progression**

**Supplementary Table 3.** Clinically evaluated progression (progressor vs. stable at 1-year follow-up) and qLRP (baseline measurements).

|  | **Odds ratio** | **95% CI** | **p-value** |
| --- | --- | --- | --- |
| Baseline pupil diameter (mm) (baseline) | 1.05 | [0.45, 2.42] | >0.9 |
| Peak constriction pupil diameter (mm) (baseline) | 0.72 | [0.18, 2.79] | 0.6 |
| Delta, relative pupillary change (percent (baseline)) | 1.04 | [0.97, 1.12] | 0.3 |
| Latency (s) (baseline) | 0.01 | - | 0.6 |
| Average constriction velocity (mm/s) (baseline) | 1.84 | [0.83, 4.35] | 0.14 |
| Maximum constriction velocity (mm/s) (baseline) | 1.50 | [0.87, 2.69] | 0.2 |
| Average dilation velocity (mm/s) (baseline) | 1.43 | [0.24, 8.72] | 0.7 |
| T75 (s) (baseline) | 1.08 | [0.54, 2.24] | 0.8 |

**Clinical Dementia Rating**

**Supplementary Table 4.** CDR SoB (1-year annualized change) and qLRP variables (baseline measurements).

|  | **Beta** | **95% CI** | **p-value** |
| --- | --- | --- | --- |
| Baseline pupil diameter (mm) (baseline) | -0.19 | [-0.57, 0.19] | 0.3 |
| Peak constriction pupil diameter (mm) (baseline) | 0.00 | [-0.62, 0.62] | >0.9 |
| Delta, relative pupillary change (percent (baseline)) | -0.03 | [-0.06, 0.00] | 0.081 |
| Latency (s) (baseline) | -0.52 | [-8.0, 7.0] | 0.9 |
| Average constriction velocity (mm/s) (baseline) | -0.12 | [-0.48, 0.23] | 0.5 |
| Maximum constriction velocity (mm/s) (baseline) | -0.14 | [-0.38, 0.11] | 0.3 |
| Average dilation velocity (mm/s) (baseline) | -0.58 | [-1.4, 0.23] | 0.2 |
| T75 (s) (baseline) | -0.17 | [-0.50, 0.16] | 0.3 |

**Supplementary Table 5.** CDR SoB (1-year annualized change, log-transformed) and qLRP variables (3-month changes). Beta values are shown for 1 unit decreases in qLRP variables.

|  | **Beta** | **95% CI** | **p-value** |
| --- | --- | --- | --- |
| Baseline pupil diameter (mm) (3-month change) | 0.34 | [-0.81, 0.13] | 0.2 |
| Peak constriction pupil diameter (mm) (3-month change) | 0.74 | [-1.8, 0.28] | 0.2 |
| Delta relative pupillary change (percent (3-month change)) | 0.02 | [-0.06, 0.03] | 0.5 |
| Latency (s) (3-month change) | -3.8 | [-5.5, 13] | 0.4 |
| Average constriction velocity (mm/s) (3-month change) | 0.42 | [-0.87, 0.02] | 0.061 |
| Maximum constriction velocity (mm/s) (3-month change) | 0.23 | [-0.52, 0.07] | 0.13 |
| Average dilation velocity (mm/s) (3-month change) | 0.32 | [-1.6, 0.99] | 0.6 |
| T75 (s) (3-month change) | 0.04 | [-0.24, 0.33] | 0.8 |

**Mini Mental-State Examination**

**Supplementary Table 6.** MMSE annualized change (1-year) and qLRP variables (3-month changes).

|  | **Beta** | **95% CI** | **p-value** |
| --- | --- | --- | --- |
| Baseline pupil diameter (mm) (3-month change) | 1.1 | [-0.35, 2.5] | 0.14 |
| Peak constriction pupil diameter (mm) (3-month change) | 1.7 | [-1.4, 4.7] | 0.3 |
| Latency (s) (3-month change) | -8.0 | [-35, 19] | 0.6 |
| Delta, relative pupillary change (percent (3-month change)) | 0.09 | [-0.03, 0.22] | 0.14 |
| Average constriction velocity (mm/s) (3-month change) | 1.2 | [-0.13, 2.5] | 0.076 |
| Maximum constriction velocity (mm/s) (3-month change) | 0.53 | [-0.35, 1.4] | 0.2 |
| Average dilation velocity (mm/s) (3-month change) | -2.3 | [-6.0, 1.4] | 0.2 |
| T75 (s) (3-month change) | 0.79 | [-0.001 1.6] | 0.054 |

***Visual [^18^F]-FDG-PET Progression***

**Supplementary Table 7.** Visual PET progression and qLRP variables (baseline).

|  | **Odds ratio** | **95% CI** | **p-value** |
| --- | --- | --- | --- |
| Baseline pupil diameter (mm) (baseline) | 1.18 | [0.39, 3.63] | 0.8 |
| Peak constriction pupil diameter (mm) (baseline) | 0.64 | [0.10, 3.99] | 0.6 |
| Delta, relative pupillary change (percent (baseline)) | 1.05 | [0.97, 1.14] | 0.2 |
| Latency (s) (baseline) | 0.08 | - | 0.8 |
| Average constriction velocity (mm/s) (baseline) | 1.42 | [0.57, 3.69] | 0.5 |
| Maximum constriction velocity (mm/s) (baseline) | 1.41 | [0.76, 2.70] | 0.3 |
| Average dilation velocity (mm/s) (baseline) | 2.08 | [0.29, 16.2] | 0.5 |
| T75 (s) (baseline) | 1.24 | [0.53, 3.00] | 0.6 |

- the 95 % CI for latency was very broad owing to the small variance in the parameter.

**Supplementary Table 8.** Visual PET progression and qLRP variables (3-month change).

|  | **Odds ratio** | **95% CI** | **p-value** |
| --- | --- | --- | --- |
| Baseline pupil diameter (mm) (3-month change) | 1.77 | [0.51, 6.46] | 0.4 |
| Peak constriction pupil diameter (mm) (3-month change) | 1.29 | [0.08, 20.6] | 0.9 |
| Delta, relative pupillary change (percent (3-month change)) | 1.08 | [0.98, 1.21] | 0.15 |
| Latency (s) (3-month change) | - | - | 0.2 |
| Average constriction velocity (mm/s) (3-month change) | 1.22 | [0.42, 3.64] | 0.7 |
| Maximum constriction velocity (mm/s) (3-month change) | 1.33 | [0.65, 2.8] | 0.4 |
| Average dilation velocity (mm/s) (3-month change) | 1.52 | [0.06, 40.8] | 0.8 |
| T75 (s) (3-month change) | 1.41 | [0.65, 3.27] | 0.4 |

- The odds ratio for latency was very high due to modeling difficulty. The reciprocal odds are shown meaning that the odds ratio reflects the increase in risk of progression resulting from a 1 unit decrease in the predictor variable.

**Confounder analysis**

**Supplementary Table 9.** Results of the logistic regression models presented in Table 2 with additional adjustment for presence of mild eye disease (model 2) and use of pupil medication (Model 3). The possible confounders are added to the fully adjusted model (model 1).

|  | Model 1 (full model w/o eye disease and medication) | | Model 2 (Mild eye disease) | | Model 3 (Pupil medication) | |
| --- | --- | --- | --- | --- | --- | --- |
|  | **Odds ratio**  **[95 % CI]** | **p-value** | **Odds ratio**  **[95 % CI]** | **p-value** | **Odds ratio**  **[95 % CI]** | **p-value** |
| Resting pupil diameter (3-month Δ) | 4.28  [1.24, 16.9] | **0.028^a^** | 4.7 [1.3,19.4] | **0.02** | 3.7  [1.04, 14.8] | 0.0504 |

# References

1. McDougal DH & Gamlin PD. Autonomic control of the eye. *Compr Physiol* **5**, 439–73 (2015).

2. Steinhauer, S. R., Condray, R. & Kasparek, A. Cognitive modulation of midbrain function: Task-induced reduction of the pupillary light reflex. *International Journal of Psychophysiology* **39**, 21–30 (2000).

3. Huang, R. & Clewett, D. The Locus Coeruleus: Where Cognitive and Emotional Processing Meet the Eye. *Modern Pupillometry* 3–75 (2024) doi:10.1007/978-3-031-54896-3_1.

4. Cazettes, F., Reato, D., Morais, J. P., Renart, A. & Mainen, Z. F. Phasic Activation of Dorsal Raphe Serotonergic Neurons Increases Pupil Size. *Current Biology* **31**, 192-197.e4 (2021).

5. Grinberg, L. T. *et al.* The dorsal raphe nucleus shows phospho-tau neurofibrillary changes before the transentorhinal region in Alzheimer̈s disease. A precocious onset? *Neuropathol Appl Neurobiol* **35**, 406–416 (2009).

6. Scinto, L. F. M. *et al.* Selective cell loss in Edinger-Westphal in asymptomatic elders and Alzheimer’s patients. *Neurobiol Aging* **22**, 729–736 (2001).

7. Scinto, L. F. M. *et al.* Focal pathology in the Edinger-Westphal nucleus explains pupillary hypersensitivity in Alzheimer’s disease. *Acta Neuropathol* **97**, 557–564 (1999).

8. Mavroudis, I. A. *et al.* Dendritic and spinal alterations of neurons from Edinger-Westphal nucleus in Alzheimer’s disease. *Folia Neuropathol* **52**, 197–204 (2014).

9. Bergamin, O. & Kardon, R. H. Latency of the pupil light reflex: Sample rate, stimulus intensity, and variation in normal subjects. *Invest Ophthalmol Vis Sci* **44**, 1546–1554 (2003).

10. Ferencova, N., Visnovcova, Z., Olexova, L. B. & Tonhajzerova, I. Eye Pupil – A Window into Central Autonomic Regulation via Emotional/Cognitive Processing. *Physiol Res* **70**, S669 (2021).

11. Gramkow, M. H., Clemmensen, F. K., Waldemar, G., Hasselbalch, S. G. & Frederiksen, K. S. Test-retest reliability and short-term variability of quantitative light reflex pupillometry in a mixed memory clinic cohort. *J Neurol Sci* **456**, 122856 (2024).
